# Genome-wide association studies identify miRNA-194 as a prognostic biomarker for gastrointestinal cancer by targeting ATP6V1F, PPP1R14B, BTF3L4 and SLC7A5

**DOI:** 10.3389/fonc.2022.1025594

**Published:** 2022-12-22

**Authors:** Pan Huang, Lingyun Xia, Qiwei Guo, Congcong Huang, Zidi Wang, Yinxuan Huang, Shanshan Qin, Weidong Leng, Dandan Li

**Affiliations:** ^1^ Department of Stomatology, Taihe Hospital and Hubei Key Laboratory of Embryonic Stem Cell Research, School of Basic Medical Sciences, Hubei University of Medicine, Shiyan, Hubei, China; ^2^ Laboratory of Tumor Biology, Academy of Bio-Medicine Research, Hubei University of Medicine, Shiyan, Hubei, China

**Keywords:** biomarker, miR-194, gastrointestinal cancer, genome-wide CRISPR-cas9 proliferation screening, oncogene, passenger gene

## Abstract

**Background:**

The dysregulated genes and miRNAs in tumor progression can be used as biomarkers for tumor diagnosis and prognosis. However, the biomarkers for predicting the clinical outcome of gastrointestinal cancer (GIC) are still scarce.

**Methods:**

Genome-wide association studies were performed to screen optimal prognostic miRNA biomarkers. RNA-seq, Ago-HITS-CLIP-seq, western blotting and qRT-PCR assays were conducted to identify target genes of miR-194. Genome-wide CRISPR-cas9 proliferation screening analysis were conducted to distinguish passenger gene and driver gene.

**Results:**

A total of 9 prognostic miRNAs for GIC were identified by global microRNA expression analysis. Among them, miR-194 was the only one miRNA that significantly associated with overall survival, disease-specific survival and progress-free interval in both gastric, colorectal and liver cancers, indicating miR-194 was an optimal prognostic biomarker for GIC. RNA-seq analysis confirmed 18 conservative target genes of miR-194. Four of them, including ATP6V1F, PPP1R14B, BTF3L4 and SLC7A5, were directly targeted by miR-194 and required for cell proliferation. Cell proliferation assay validated that miR-194 inhibits cell proliferation by targeting ATP6V1F, PPP1R14B, BTF3L4 and SLC7A5 in GIC.

**Conclusion:**

In summary, miR-194 is an optimal biomarker for predicting the outcome of GIC. Our finding highlights that miR-194 exerts a tumor-suppressive role in digestive system cancers by targeting ATP6V1F, PPP1R14B, BTF3L4 and SLC7A5.

## Introduction

The gastrointestinal tract (GIT), also known as digestive tract, is the tract from the mouth to the anus which includes all the organs of the digestive system in humans and other animals (1). Gastrointestinal cancer (GIC) refers to malignant conditions of the GIT and accessory organs of digestion, including the esophagus, stomach, biliary system, pancreas, small intestine, large intestine, rectum and anus (2). Gastric cancer, colorectal cancer and liver cancer, accounted for the vast majority of GIC, are the major global medical and economic burdens (2, 3). Although current treatments for GIC have been greatly improved, the prognosis of GIC remains unsatisfied to date due to the inconvenience of early diagnosis of GIC patients (4–6). Besides, the molecular mechanisms underlying GIC progression remains unclear (7–9). Hence, it is urgent and necessary to explore novel potential biomarkers and their molecular mechanisms to better understand the pathophysiology of GI tumors.

Mature microRNAs (miRNA) are a group of small non-coding RNAs with 19-24 nucleotides located in the human genome (10). It is well known that miRNA usually negatively regulates the expression of target genes at post-transcription level by binding to the specific regions in the 3’-untranslated regions (3’-UTRs) of target transcripts (11–13). Increasing evidence has confirmed that miRNAs play essential roles in tumorigenesis and metastasis by targeting oncogenes and tumor suppressor genes (14–16). Depending on the regulation of target genes, miRNAs play key roles in multiple cellular events, including epithelial–mesenchymal transition (EMT), cell invasion, cell apoptosis, cell proliferation and so on (13, 17). Additionally, due to their stability, miRNAs can also serve as prognostic markers for predicting clinical outcomes (18–20). Therefore, the identification of tumor-associated miRNAs as biomarkers for early tumor detection, prognostication and treatment is of great importance.

**Figure 1:**
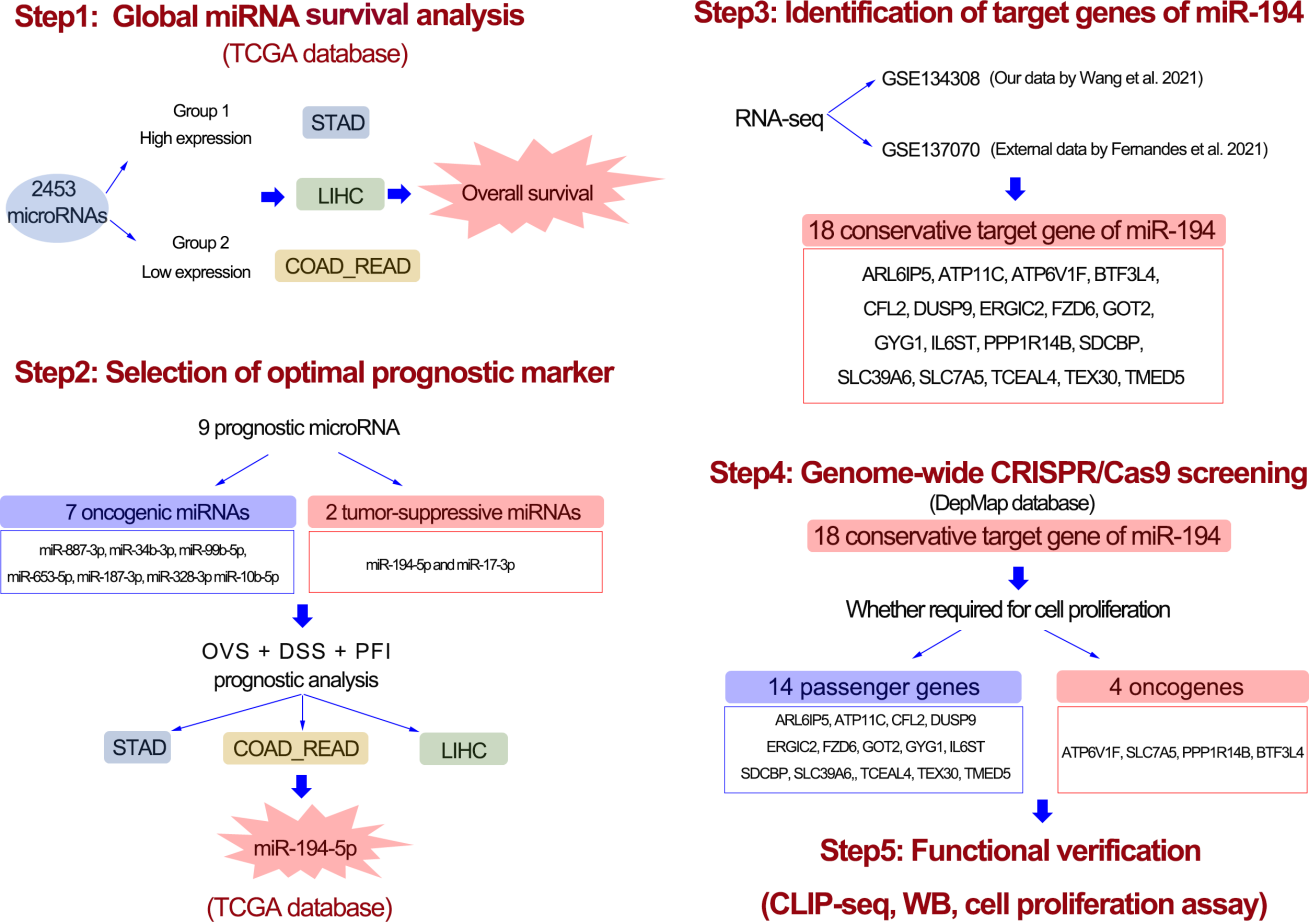
The workflow of entire study. First, with the aid of global microRNA expression analysis, a total of 9 prognostic miRNAs for GIC were identified. Second, after analysis of disease-specific survival and progress-free interval, miR-194 turn out to be an optimal prognostic biomarker for GIC. Third, 18 potential target genes greatly downregulated by miR-194 were screened using transcriptome sequencing. Forth, genome-wide CRISPR-CAS9 knockout screening identified 4 miR-194 target genes required for cell proliferation. Fifth, the Ago-HITS-CLIP-seq, qRT-PCR, immunoblotting and cell proliferation assays together confirmed miR-194 inhibited GIC proliferation by targeting ATP6V1F, BTF3L4, PPP1R14B and SLC7A5.

In this study, we designed a workflow for screening prognostic marker miRNAs in GIC (**Figure 1**). Firstly, we used global miRNA expression analysis method to identify the potential miRNAs simultaneously associated with gastric cancer, colorectal cancer and liver cancer. A total of 9 prognostic miRNAs were identified in GIC, including 2 miRNAs with favorable prognosis and 7 miRNAs with poor prognosis. Among them, only miR-194-5p showed a significant correlation with overall survival, disease-specific survival and progress-free survival in GIC patients. Thus, we selected miR-194 to further explore its tumor suppressive mechanism. With the aid of RNA-seq and Ago-HITS-CLIP-seq analysis and Genome-wide CRISPR/Cas9 proliferation screening system, we confirmed that miR-194 inhibited GIC cells proliferation by targeting ATP6V1F, SLC7A6, PPP1R14B and BTF3L4.

## Results

### Identification of prognostic biomarker miRNAs for GIC

**Figure 2:**
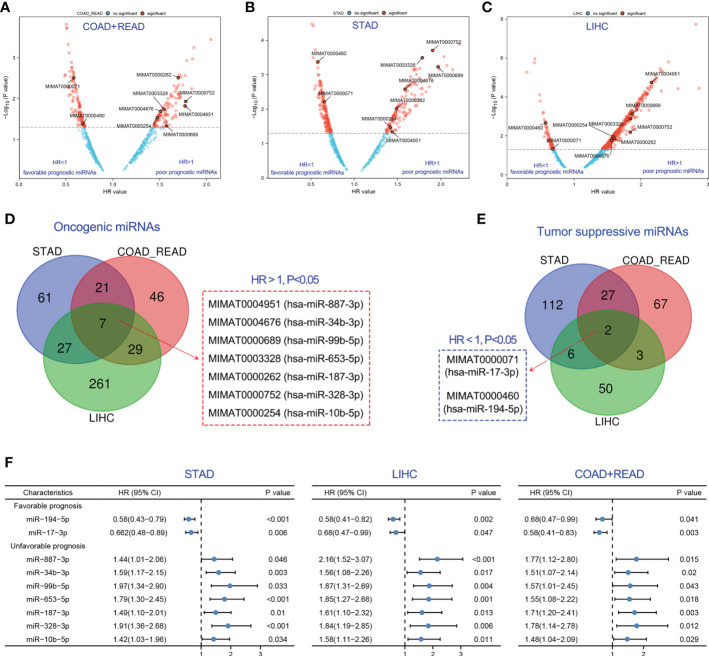
Identification of optimal prognostic miRNAs for GIC. **(A-C)** Exploration of prognostic miRNAs for predicting the overall survival of colorectal, gastric and liver cancer. **(D)** 7 prognostic miRNAs were associated with poor prognosis of GIC. **(E)** 2 prognostic miRNAs were associated with poor prognosis of GIC. **(F)** Forest plot showing the association of the 9 prognostic miRNAs of GIC with overall survival prognosis in gastric cancer (STAD), colorectal cancer (COADREAD) and liver cancer (LIHC).

Various miRNAs possess a significant correlation with cancer prognosis. Herein, we developed a strategy to identify prognostic biomarker miRNAs in gastric (STAD), colorectal (COAD and READ), and liver cancers (LIHC) using the TCGA database. The workflow for this study was shown in **Figure 1**. Firstly, global miRNA survival analysis was conducted to reveal the prognostic values of each miRNA in GIC patients. Hazard ratio (HR) value greater than 1 and p value less than 0.05 means miRNA is significantly associated with poor tumor prognosis; HR value less than 1 and p value less than 0.05 means miRNA is significantly associated with good tumor prognosis. With this approach, we identified 202 miRNAs significantly associated with colorectal cancer prognosis, including 103 poor prognostic miRNAs and 99 favorable prognostic miRNAs (**Figure 2A**). 263 miRNAs were significantly correlated with the prognosis of gastric cancer (GC), including 116 poor prognostic miRNAs and 147 favorable prognostic miRNAs (**Figure 2B**). In liver cancer, 385 miRNAs were significantly correlated with patients’ prognosis, including 324 poor prognostic miRNAs and 61 favorable prognostic miRNAs (**Figure 2C**). After taking the intersection, 7 poor prognostic miRNAs of GIC were identified, including miR-887-3p, miR-34b-3p, miR-99b-5p, miR-653-5p, miR-187-3p, miR-328-3p and miR-10b-5p (**Figure 2D**). Similarly, 2 favorable prognostic miRNAs of GIC were identified, including miR-194-5p and miR-17-3p (**Figure 2E**). The forest plot was performed to show the prognostic associations of these 9 miRNAs with gastric, colorectal, and liver cancers in detail (**Figure 2F**).

### MiR-194 can serve as a biomarker of GI tissue and a prognostic marker of GIC

**Figure 3:**
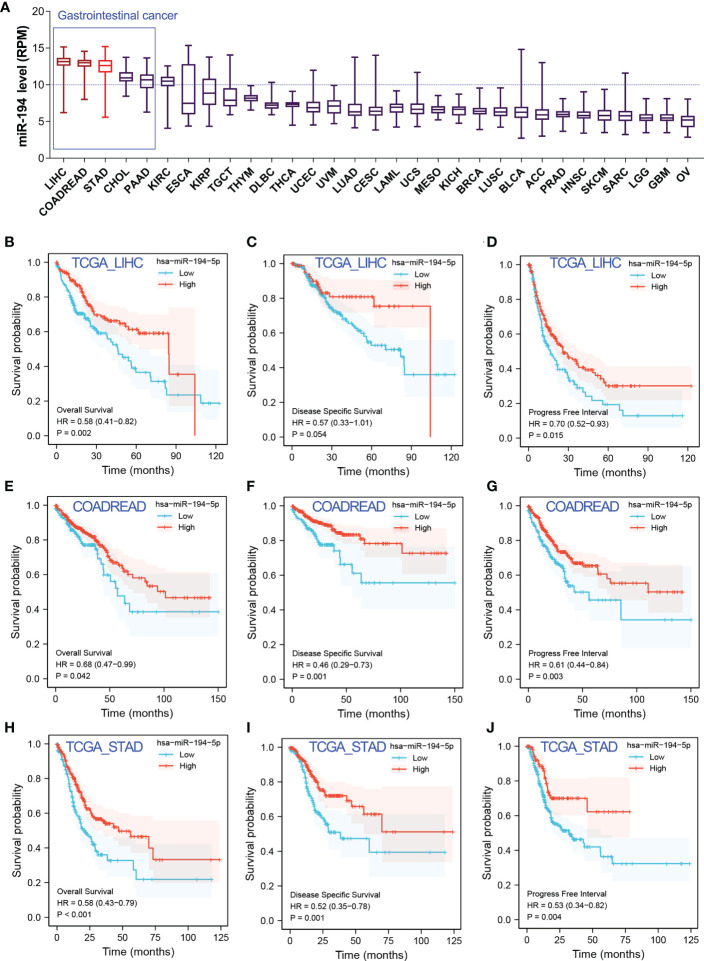
MiR-194 was identified to be an optimal prognostic biomarker miRNA for GIC. **(A)** The expression pattern of miR-194 in pan-cancer showed that miR-194 was highly expressed in GIC. **(B-D)** Low expression of miR-194 was associated with shorter time of overall survival (OVS), disease-specific survival (DSS) and progress-free interval (PFI) in patients with liver cancer (LIHC). **(E-G)** Low expression of miR-194 was associated with shorter time of OVS, DSS and PFI survival in patients with colorectal cancer (COADREAD). **(H-J)** Low expression of miR-194 predicted poor OVS, DSS and PFI survival in patients with stomach cancer (STAD).

Next, the optimal prognostic biomarker was selected for sequential analysis. We chose miR-194 for the following two reasons. The first reason was that miR-194 was a GIT/GIC-enriched microRNA. Our pan-cancer analysis revealed that miR-194 has the highest expression in liver cancer, colorectal cancer, stomach cancer compared to the expression levels in other tumors (**Figure 3A**), suggesting that miR-194 can be used as a biomarker for GIC. Another important reason was that miR-194 was a prognostic marker for GIC, since its expression was significantly correlated with favorable overall survival (OVS), disease-specific survival (DSS) and progress-free interval (PFI) in STAD, COADREAD and LIHC (**Figures 3B–J**).

Most miRNAs are located in the gene body of host genes. Thus, miRNAs and its host genes usually share regulatory elements, primary transcripts, and have similar expression patterns. LncRNA MIR194-2HG is a host gene of miR-194. The expression pattern analysis showed that MIR194-2HG selectively expressed in liver, colorectal and stomach (**Figure S1A**), implied that miR-194 highly expressed in GIT. However, as the host gene of the miR-17-3p, MIR17-HG was highly expressed in leukemia, lowly expressed in GIT/GIC (**Figure S1B**).

Our survival analysis has implied that miR-17-3p plays a tumor-suppressive role in GIC, since high expression of miR-17 predicted favorable overall survival in GIC. Controversially, it has been reported that miR-17-3p functioned oncogenic roles in liver cancer and gastric cancer (21–23). In addition, although miR-17-3p predicted favorable overall survival in GIC, we also observed inconsistent evidence that miR-17-3p predicted poor DSS survival in LIHC, and showed no significant association with PFI survival in COADREAD and LIHC (**Figure S1C-E**). Consistently, we previously reported that miR-194 functioned as a tumor suppressor gene in gastric cancer (4). These evidence together showed that miR-17-3p was not a stable prognostic biomarker for GIC, while miR-194 can serve as a stable prognostic marker in GIC.

### Identification of the downregulated genes of miR-194 by RNA-seq analysis

It’s well known that miRNA negatively modulates target gene expression by miRNA-mRNA interaction. To better understand the biological function of miR-194 in cancer, two independent RNA-seq data of miR-194 overexpression, including GSE137070 and GSE134308, were further analyzed to identify potential target genes of miR-194. Rayzel and colleagues have uploaded the RNA-seq data of GSE137070 on miR-194 overexpression in the prostate cancer cell line 22Rv1 (24). While, we had previously uploaded the RNA-seq data of GSE134308 on miR-194 overexpression in the gastric cancer cell lines SGC7901 and BGC823 (4).

**Figure 4:**
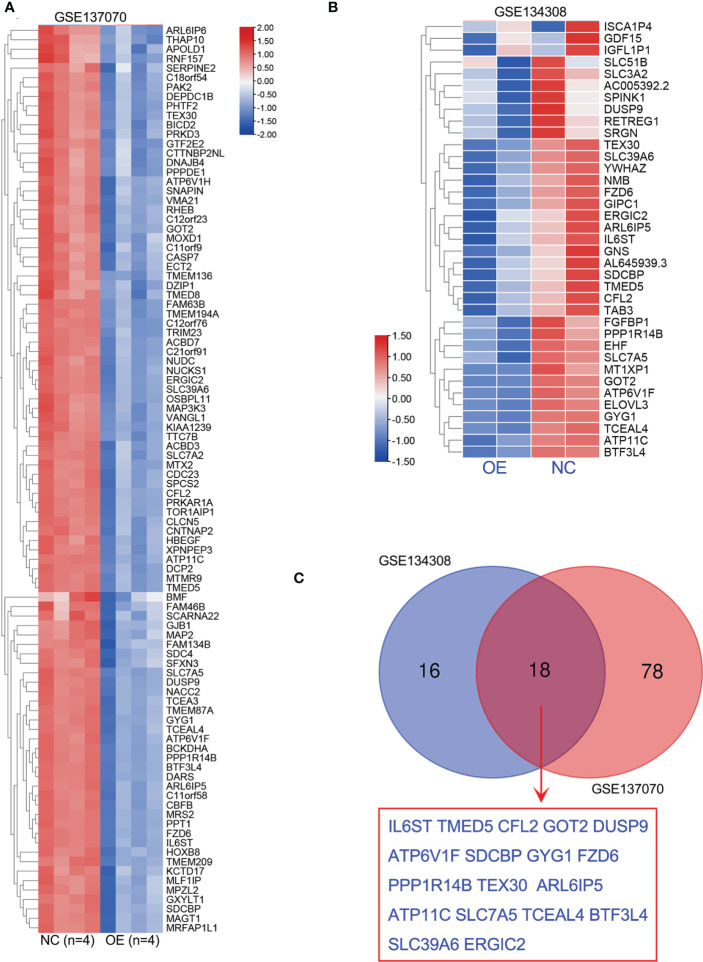
Identification of potential target genes of miR-194 by RNA sequencing. **(A)** A total of 96 genes greatly downregulated by miR-194 in GSE137070 dataset were screened (log2FC<-1). **(B)** After transfection with miR-194 mimics in GC cell lines SGC7901 and AGS, RNA-seq studies were conducted to explore target genes of miR-194 (GSE134308). A total of 34 genes greatly downregulated by miR-194 (log2FC<-0.8) were screened according to RNA-seq analysis. **(C)** A total of 18 genes greatly downregulated by miR-194 in both GSE137070 and GSE134308 datasets were screened. **P < 0.01.

Firstly, we obtained the RNA-seq data of GSE137070 from GEO dataset and analyzed the downregulated genes by miR-194 overexpression. A total of 96 genes with count value more than 100 and log2FC less than -1 were shown in the heatmap (**Figure 4A**). Next, we additionally analyzed our RNA-seq data of miR-194 overexpression in GC cell lines. A total of 34 genes with log2FC less than -0.8 in both two GC cell lines were shown in the heatmap (**Figure 4B**). After taking the intersection, we found a total of 18 genes were conserved negatively regulated by miR-194, including ARL6IP5, ATP11C, ATP6V1F, BTF3L4, CFL2, DUSP9, ERGIC2, FZD6, GOT2, GYG1, IL6ST, PPP1R14B, SDCBP, SLC39A6, SLC7A5, TCEAL4, TEX30, TMED5 (**Figure 4C**).

### Genome-wide CRISPR/Cas9 proliferation screening assay confirmed ATP6V1F, SLC7A5, PPP1R14B and BTF3L4 were required for tumor cell proliferation

Increasing studies have reported that miR-194 suppresses tumor cell proliferation. For an example, our previous study has reported that miR-194 overexpression largely inhibited the cell proliferation in GC cell lines (4). The current studies have identified 18 putative target genes of miR-194 by RNA-seq analysis. However, it remains unclear which genes miR-194 targets to suppress tumor cell growth.

To understand the contribution of these 18 target genes of miR-194 to cell proliferation, we analyzed the Genome-wide CRISPR/Cas9 proliferation screening data in Depmap dataset. According to the DEMETER2 method in this database, the lower Chronos dependency score, the greater probability that the gene is an oncogene and required for the cell proliferation of given cell line. In contrast, the higher Chronos dependency score, the greater probability that the gene is a tumor suppressor gene that negatively regulates cell proliferation of given cell line. While, if the closer the Chronos dependency score is to 0, the greater probability that the gene is a passenger gene that has no obvious effects on cell proliferation of given cell line. It is widely believed that oncogene or tumor suppressor gene is the cause of tumorigenesis, while the passenger gene are the resultant effects after tumorigenesis.

**Figure 5:**
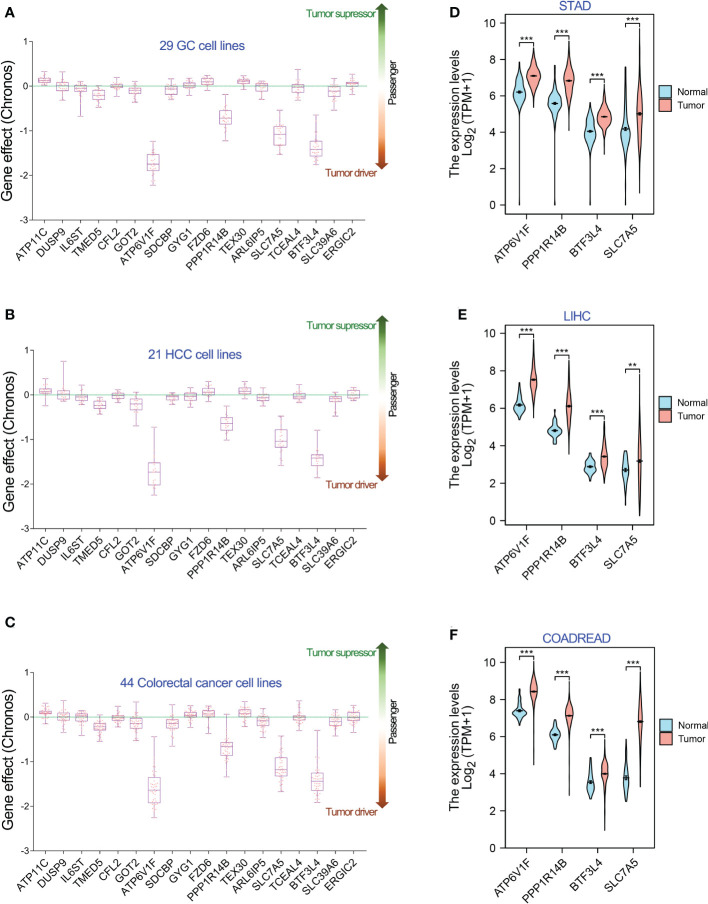
Genome-wide CRISPR-cas9 knockout screening identifies 4 genes required for cell proliferation of GIC cell lines. **(A-C)** In 18 genes greatly downregulated by miR-194, 4 genes, including ATP6V1F, PPP1R14B, SLC7A5 and BTF3L4, were required for cell proliferation in 26 gastric cancer cell lines, 21 hepatology cancer cell lines and 44 colorectal cancer cell lines. **(D-F)** Gene expression analysis showed that ATP6V1F, PPP1R14B, SLC7A5 and BTF3L4 were overexpressed in gastric cancer (STAD), colorectal cancer (COADREAD) and liver cancer (LIHC). **P < 0.01. ***P < 0.001.

Hence, we analyzed the Chronos dependency score of the 18 target genes of miR-194 using Depmap dataset. The results showed that only four genes, including ATP6V1F, PPP1R14B, BTF3L4 and SLC7A5, were oncogenes that are required for cell proliferation of stomach cancer, liver cancer and colorectal cancer (**Figures 5A-C**). The other 14 genes were passenger genes of GIC occurrence. Consistent with the results of Genome-wide CRISPR/Cas9 proliferation screening assay, ATP6V1F, PPP1R14B, BTF3L4 and SLC7A5 were significantly upregulated in STAD, LIHC and COADREAD (**Figures 5D-F**).

### MiR-194 negatively regulates ATP6V1F, PPP1R14B, BTF3L4 and SLC7A5 by binding to their mRNAs

The genes that are negatively regulated by miRNAs can be divided into two categories, one is the genes that are directly targeted by miRNAs, and the other is the genes that are indirectly regulated by miRNAs. It is known that miRNAs can directly regulate gene expression by binding to target mRNA and affecting mRNA stability. To figure out how miR-194 negatively regulates the expression of ATP6V1F, PPP1R14B, BTF3L4 and SLC7A5, we firstly identified that the seed sequence binding sites of miRNA-194 existed within the mRNA of all the 18 genes by searching the miRcode dataset (**Table 1**). The result implied that miR-194 might directly down-regulate these 18 genes by binding to their mRNAs.

**Table 1:** The predicted binding sites of miR-194-5p were analyzed in the 18 potential target genes greatly downregulated by miR-194 using the miRcode online web tool.

Gene symbols	Number of sites	Type	Position
ARL6IP5	3	7-mer-A1	3’UTR
ATP11C	2	7-mer-A1; 8-mer	CDS (1); 3’UTR (1)
ATP6V1F	1	7-mer-A1	3’UTR
BTF3L4	3	7-mer-A1 (2); 7-mer-m8 (1)	3’UTR
CFL2	2	7-mer-m8	3’UTR
DUSP9	1	7-mer-m8	3’UTR
ERGIC2	1	8-mer	3’UTR
FZD6	2	7-mer-A1; 8-mer	CDS (1); 3’UTR (1)
GYG1	1	8-mer	3’UTR
IL6ST	4	7-mer-A1 (3); 8-mer (1)	3’UTR
PPP1R14B	1	8-mer	CDS
SDCBP	1	8-mer	3’UTR
SLC39A6	1	8-mer	3’UTR
GOT2	1	8-mer	3’UTR
SLC7A5	1	8-mer	3’UTR
TCEAL4	1	8-mer	3’UTR
TEX30	2	7-mer-A1 (3); 8-mer (1)	3’UTR
TMED5	6	7-mer-A1 (3); 7-mer-m8 (2);8-mer (1)	3’UTR

**Figure 6:**
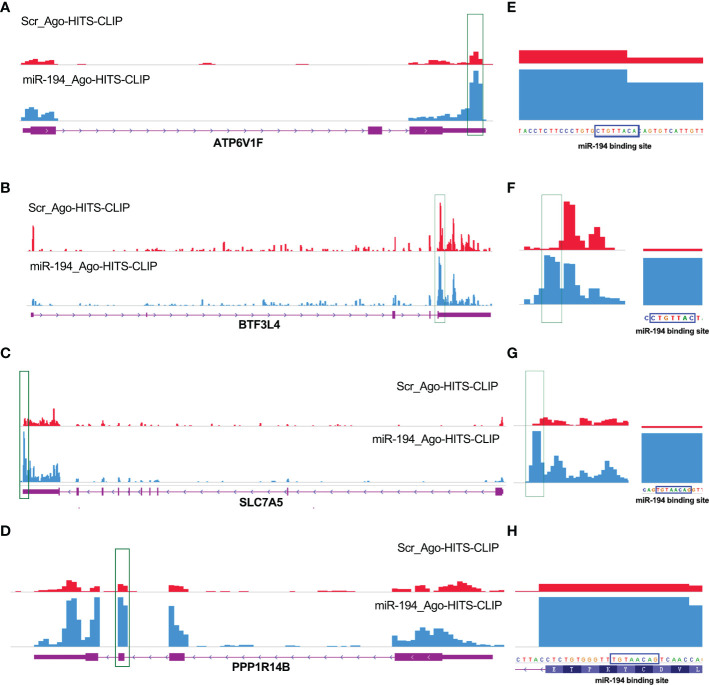
Ago-HITS-CLIP-seq analysis identified ATP6V1F, PPP1R14B, SLC7A5 and BTF3L4 as target genes of miR-194. **(A-D)** An obvious binding peak of miR-194 was present within the mRNA of ATP6V1F, PPP1R14B, SLC7A5 and BTF3L4, respectively. The statistical results were shown on the right panel. The binding site of miR-194 and PPP1R14B gene is located in in the coding region. While the binding site of miR-194 and other three genes is located in the 3’ untranslated region. **(E-H)** There was an obvious miR-194 seed sequence binding site inside the miR-194 binding peaks identified by Ago-HITS-CLIP-seq analysis. The binding sites of miR-194 in 4 genes identified by Ago-HITS-CLIP-seq analysis are highly consistent with the predicted results of bioinformatics analysis.

To confirm the interaction between miR-194 and the mRNA of ATP6V1F, PPP1R14B, BTF3L4 and SLC7A5, we analyzed the Ago-HITS-CLIP-seq data (GSE137071) of miR-194 (24). The Ago-HITS-CLIP-seq data contains the binding signal of miR-194 in various transcripts. After calling peak, an obvious binding peak for miR-194 existed in the transcripts of ATP6V1F, PPP1R14B, BTF3L4 and SLC7A5 (**Figures 6A–D**). Additionally, after amplifying the binding peak of miR-194, there was an obvious miR-194 seed sequence binding site within the binding peak that are consistent with the predicted results, suggesting miR-194 can directly bind on the transcripts of those genes (**Figures 6E–H**). Given that microRNA negatively regulates gene expression by interacting with their mRNA to affect mRNA stability, ATP6V1F, PPP1R14B, BTF3L4 and SLC7A5 were putative target genes of miR-194.

### MiR-194 inhibits GIC cell proliferation by targeting ATP6V1F, PPP1R14B, BTF3L4 and SLC7A5

**Figure 7:**
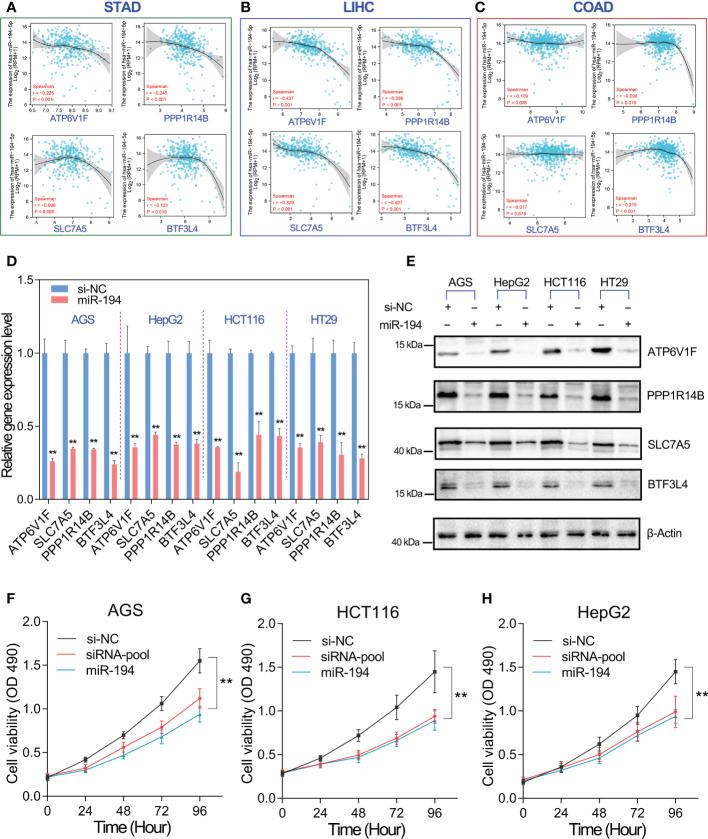
MiR-194 inhibited GIC cell proliferation by targeting ATP6V1F, PPP1R14B, SLC7A5 and BTF3L4. **(A-C)** The expression of miR-194 was negatively associated with the expression of ATP6V1F, PPP1R14B, SLC7A5 and BTF3L4 in gastric cancer (STAD), colorectal cancer (COADREAD) and liver cancer (LIHC). **(D)** The expression of ATP6V1F, PPP1R14B, SLC7A5 and BTF3L4 were determined after overexpression of miR-194 in GIC cell lines. **(E)** Overexpression of miR-194 greatly downregulated the protein level of ATP6V1F, PPP1R14B, SLC7A5 and BTF3L4. **(F-H)** Cell proliferation assays showed that overexpression of miR-194 had similar effects on cell proliferation as siRNA pools containing siRNAs targeting ATP6V1F, PPP1R14B, SLC7A5, and BTF3L4. **P < 0.01.

We have confirmed that miR-194 directly downregulated the expression of ATP6V1F, PPP1R14B, BTF3L4 and SLC7A5. That means the expression of miR-194 should be negatively correlated with their expression in cells and tissues. To verify this possibility, the gene expression correlation between miR-194 and target genes was analyzed. The gene expression correlation analysis showed that miR-194 expression showed a negative correlation with the expression of ATP6V1F, PPP1R14B, BTF3L4 and SLC7A5 in gastric cancer, liver cancer and colorectal cancer (**Figures 7A–C**).

On the other hand, we furthermore analyzed the effect of miR-194 overexpression on the mRNA and protein levels of ATP6V1F, PPP1R14B, BTF3L4 and SLC7A5 in different GIC cell lines. The results showed that mRNA levels of ATP6V1F, PPP1R14B, BTF3L4 and SLC7A5 were strongly downregulated by miR-194 in different GIC cell lines (**Figure 7D**). Consistent with qRT-PCR assay, western blotting assay also showed that miR-194 overexpression greatly downregulated the protein levels of ATP6V1F, PPP1R14B, BTF3L4 and SLC7A5 in different GIC cell lines (**Figure 7E**). More importantly, cell proliferation assay showed that the inhibitory effect of miR-194 on GIC cell proliferation was nearly equivalent to that of a pool of siRNA targeting 4 genes, including ATP6V1F, PPP1R14B, BTF3L4 and SLC7A5 (**Figures 7F-H**). These results suggested that miR-194 mainly inhibited GIC cell proliferation by directly targeting ATP6V1F, PPP1R14B, BTF3L4 and SLC7A5.

## Discussion

Non-coding RNA (ncRNA) constitute the majority of the human transcriptome, including a class of small ncRNAs (including miRNAs and piRNAs), lncRNAs and circular RNAs (25–27). Increasing studies have reported that miRNAs play critical roles in tumorigenesis and metastasis and could serve as diagnostic and prognostic biomarkers for cancer-related patients (28). In this work, our integrated analysis comprehensively identified 9 prognostic biomarkers for GIC, including miR-194-5p, miR-17-3p, miR-887-3p, miR-34b-3p, miR-99b-5p, miR-653-5p, miR-187-3p, miR-328-3p and miR-10b-5p. Among them, only miR-194 possessed significant association with OVS, DSS and PFI survival of GIC, suggesting miR-194 could serve as optimal prognostic biomarker for GIC.

Increasing evidence has shown that miR-194-5p functioned as a tumor suppressor gene in cancers (29). Given miR-194-5p and its host gene highly expressed in GIT tissues, miR-194 may play a critical role in the tumorigenesis of GIC. Consistent with our integrated analysis, miR-194 has been reported to play tumor suppressive roles in GIC, including gastric cancer, liver cancer, colorectal cancer and pancreas cancer (30–34).

However, these studies regarding miR-194 in cancers have several limitations. The first restriction is that these studies did not comprehensively explore the potential target genes of miR-194. The potential target genes of miRNAs are generally highly diverse, they often need to be identified by transcriptome sequencing, but not only by depending on bioinformatics prediction (35). The second restriction is that these studies did not focus on the binding preference of miRNA to target genes, so that the major pathways through which miR-194 exerts tumor suppressor effects remains unknown. Due to the preference of miRNAs to target genes, there must be some target genes that are strongly regulated by miRNAs (36–38). The third restriction is that these studies did not focus on the contribution of target genes to tumor progression. The target genes of miR-194 can be divided into oncogenes and passenger genes. In fact, dysregulation of oncogenes is the key cause of tumorigenesis, while the dysregulation of the passenger gene are the resultant effects of tumorigenesis (39). These limitations greatly impair our understanding of the tumor suppressor role of miR-194.

Transcriptome sequencing is the suitable approach to comprehensively understanding of potential target genes of miR-194. According to RNA-seq data, we can assess fold change of gene expression for each gene, thereby understanding the preference of miR-194 to target genes. Genome-wide CRISPR/Cas9 proliferation screening can help us understand the contribution of each gene to tumor progression. To figure out the key signaling axis mediated by miR-194, two independent RNA-seq data of miR-194 were analyzed. A total of 18 genes that greatly downregulated by miR-194 were identified. Genome-wide CRISPR/Cas9 proliferation screening assay confirmed that 4 genes, including ATP6V1F, PPP1R14B, BTF3L4 and SLC7A5, were required for GIC cell proliferation. Our subsequent analysis further demonstrated these four genes play oncogenic roles in GIC. Consistent with our results, ATP6V1F, PPP1R14B and SLC7A5 were reported to play oncogenic roles in colorectal cancer progression (40–42).

In conclusion, miR-194 is an optimal biomarker for predicting the clinical outcomes pf GIC patients. Transcriptome sequencing and Genome-wide CRISPR/Cas9 proliferation screening confirmed that miR-194 play tumor suppressive roles in GIC mainly by targeting ATP6V1F, BTF3L4, PPP1R14B and SLC7A5.

## Materials and methods

### Global miRNA survival analysis in GIC

MicroRNA-Seq data of GIC samples and the correlated clinical information were downloaded from the Cancer Genome Atlas (TCGA). Expression level of each miRNA was calculated from log2 of its TPM value. Each miRNA was divided into high and low groups according to its expression level. The most significant cut-off value is used as the best cutoff to separate the input data into two groups, thereby obtaining the most significant survival curve (43). The overall survival analysis of global miRNA in GIC was shown in volcano plot using HR value and p value of the most significant survival curve. The HR and p values of per miRNA were calculated using survminer R package (44). The filter conditions were set to p < 0.05.

### RNA-seq analysis

Our previous study has obtained the RNA-seq data of GSE134308 in gastric cancer cell lines (4). The RNA-seq data of GSE137070 was downloaded from GEO dataset. GSE137070 contains the transcriptome sequencing data of miR-194 overexpression in prostate cancer cell line 22Rv1 (24). The strongly downregulated genes by miR-194 was screened by RNA-seq analysis. The filter conditions were set to log2FC value < -0.8 in GSE134308 and the log2FC value < -0.8 in GSE137070.

### Analysis of genome-wide CRISPR/Cas9 proliferation screening data in GIC cell lines

One lakh seventy thousand six hundred and thirty-four genes knockout data for 563 cell lines of 27 primary diseases were obtained from the DepMap (22Q2 version) database (https://depmap.org/portal/). In this database, Chronos dependency score, based on data from a cell depletion assay, was used to represent the changes of cell growth after interest genes were knocked out. A score of 0 indicates a gene is not essential for cell growth; correspondingly −1 is comparable to the median of all pan-essential genes. The lower the score, the more likely the gene is an oncogene. The higher the score, the more likely the gene is a tumor suppressor gene.

### Ago-HITS-CLIP-seq analysis

The miR-194 binding site in relevant mRNA was predicted and analyzed using miRcode online web tool. The miR-194 binding site in relevant mRNA was further validated using Ago-HITS-CLIP-seq method. The Ago-HITS-CLIP-seq data regarding miR-194 (GSE137071) was downloaded from GEO dataset (24). The binding information of miR-194 for each transcript was visualized by Integrative Genomics Viewer (IGV) software.

### Cell culture and transfection

The human cancer cell lines used in this study were purchased from the Shanghai Cell Bank of Chinese Academy of Sciences (Shanghai, China). For siRNA transfection, the miR-194 mimics were synthesized by Genepharma Company (Shanghai, China). GIC cell lines were transfected with siRNAs (final concentration, 50 nM) by Lipofectamine 2000 (Invitrogen) according to the manufacturer’s instructions. At 72h post-transfection, cells were harvested for western blotting and qRT-PCR assay.

### Cell proliferation assays

Cells transfected with miR-194 mimics for 24 h were reseeded in 96-well plates at 2,000 cells/well in a final volume of 100 μL and cultured for 4 days. The effects of miR-194 on cell proliferation were determined with CCK-8 assay every 24 hours. Subsequently, 10 μL of CCK-8 solution (Biosharp, China) were added into each well and incubated for 2 hours. Optical density was measured at a wavelength of 490 nm by an automatic microplate reader (Bio Tek, USA). Triplicate wells were assayed for each experiment, and three independent experiments were performed. Data were expressed as the OD490 mean ± S.D.

### Quantitative RT-PCR assay

The qRT-PCR assay was performed as previously described (45). Briefly, GIC cells were grown in 6-well plates and transfected with siRNAs. After 48 hours, total RNA was extracted using Trizol reagent (Invitrogen, USA). The qPCR analysis was performed on Bio-Rad CFX Manager 3.1 real-time PCR system. The primer of PPP1R14B: ACACCCCAGAAGAAGTGACG and CCCCAAAAGTGGAAAACTGA; The primer of ATP6V1F: TGATTTCCAATTCCCTGCTC and GATGGTCAGGCTTCCTGTTC; The primer of BTF3L4: CCGAAGCTCATGGTTCTAGG and AGCTGGCTCCTGTGTAATGG; The primer of SLC7A5: TGTGATCCTTGGTGCTGTGT and GGGACGTTTGTCAGTGGAGT.

### Western blotting assay

The western blotting assay was performed as previously described (46). After 72h transfected with miRNA, GIC cells were lysed in RIPA buffer added 1 mM PMSF. Approximately 100 μg of total protein was electropharesed through 10% SDS polyacrylamide gels and were then transferred to a PVDF membrane (Millipone). After blocking with 5% skimmed milk at 4°C for 1h, the membrane was incubated with ATP6V1F antibody (1:1000, Proteintech, 17725-1-AP), BTF3L4 antibody (1:1000, Proteintech, 16500-1-AP), SLC7A5 antibody (1:2000, Abclonal, A2833), PPP1R14B antibody (1:2000, Abclonal, A4677) and β-Actin (1:10000, Proteintech, 20536-1-AP) at 4°C overnight.

### Statistical analysis

Survival analysis were calculated using the Kaplan–Meier method, and differences between the curves were analyzed using the log-rank test. The Pearson Correlation analysis was used to test the correlation between the two groups of data. The qPCR and cell proliferation assay were used unpaired t-test or one-way ANOVA test. The P values for gene expression analysis in normal and tumor tissues were estimated using Mann–Whitney nonparametric test. For all experiments, a minimum of triplicates per group and repetition of at least three times was applied to achieve reproducibility. All tests with p values less than 0.05 considered statistically significant.

## Data availability statement

The datasets presented in this study can be found in online repositories. The names of the repository/repositories and accession number(s) can be found below: https://www.ncbi.nlm.nih.gov/, GSE134308.

## Ethics statement

The study is approved by the Ethics Committee of Hubei University of Medicine (2018-TH-035). All participants provided written informed consent. The study protocol conformed to the ethical guidelines of the 1975 Declaration of Helsinki.

## Author contributions

SQ and WL designed experiments, offered direction and help on the whole project. DL, QG, ZW, PH, CH, and YH conducted the experiments, analyzed the results. SQ, DL, and CH performed bioinformatics analysis. SQ and DL drafted the manuscript. LX, WL, SQ, and DL reviewed the manuscript and made significant revisions on the drafts. All authors read and approved the final manuscript.

## Supplementary material

Supplementary Figure 1 The Expression pattern and prognostic analysis of MIR17HG. (A, B) The expression pattern of MIR17HG and MIR194-2HG in pan-tissue (GTEx database) and pan-cancer (TCGA database). (C) MIR17HG predicted favorable OVS, DSS and PFI survival in STAD (gastric cancer). (D) MIR17HG predicted favorable OVS, DSS and PFI survival in COAD and READ (colorectal cancer). (E) MIR17HG predicted favorable OVS survival, but has no significant correlation with the DSS and PFI survival in LIHC (liver cancer).
