# Supplementary figures and images for: Genome-wide association studies identify miRNA-194 as a prognostic biomarker for gastrointestinal cancer by targeting ATP6V1F, PPP1R14B, BTF3L4 and SLC7A5
Source: Front Oncol. 2022 Dec 22;12:1025594. doi: 10.3389/fonc.2022.1025594 (PMC9815773; doi:10.3389/fonc.2022.1025594)

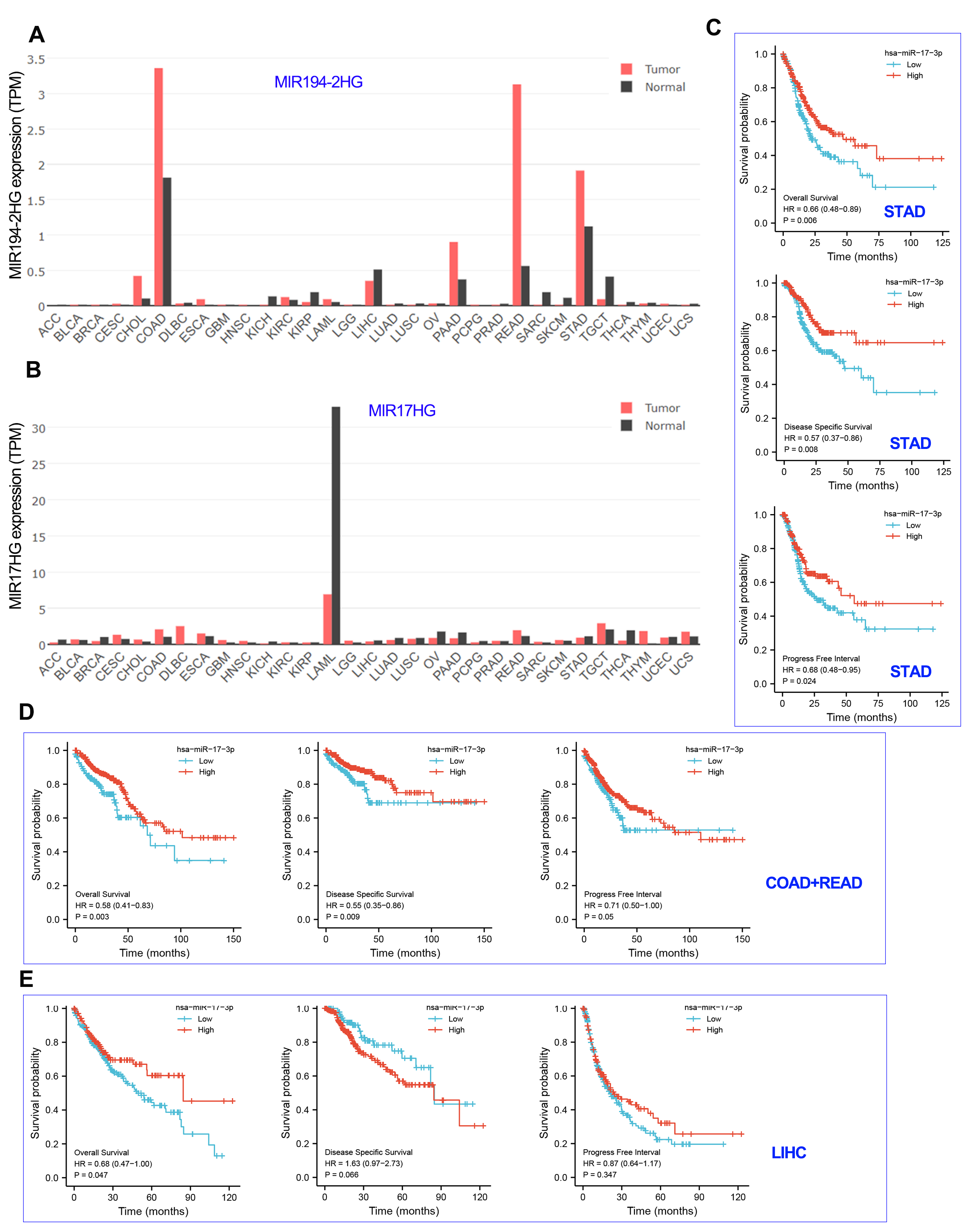

Supplement: Supplementary Figure 1 — The Expression pattern and prognostic analysis of MIR17HG. (A, B) The expression pattern of MIR17HG and MIR194-2HG in pan-tissue (GTEx database) and pan-cancer (TCGA database). (C) MIR17HG predicted favorable OVS, DSS and PFI survival in STAD (gastric cancer). (D) MIR17HG predicted favorable OVS, DSS and PFI survival in COAD and READ (colorectal cancer). (E) MIR17HG predicted favorable OVS survival, but has no significant correlation with the DSS and PFI survival in LIHC (liver cancer). [file Image_1.tif]
